# Assessment of the vitamin D status and its determinants in young healthy students from Palestine

**DOI:** 10.1017/jns.2023.25

**Published:** 2023-03-17

**Authors:** Janina Susann Lenz, Nathan Tintle, Felix Kerlikowsky, Manal Badrasawi, Rana Zahdeh, Radwan Qasrawi, Andreas Hahn, Jan Philipp Schuchardt

**Affiliations:** 1 Institute of Food Science and Human Nutrition, Leibniz University Hannover, Am Kleinen Felde 30, Hannover 30167, Germany; 2 Department of Population Health Nursing Science, College of Nursing, University of Illinois – Chicago, Chicago, IL, USA; 3 Department of Nutrition and Food Technology, Faculty of Agriculture and Veterinary Medicine, An-Najah National University, Nablus, West Bank, Palestine; 4 Department of Chemistry and Applied Sciences, College of Applied Sciences, Palestine Polytechnic University, Hebron, West Bank, Palestine; 5 Department of Computer Science, Al-Quds University, Jerusalem, Palestine; 6 Department of Computer Engineering, Istinye University, Istanbul, Turkey

**Keywords:** Calcidiol, Middle East, Vitamin D deficiency, Vitamin D status predictors

## Abstract

The global prevalence of vitamin D deficiency is high. Poor vitamin D status, especially in women, has been reported in several countries in the Middle East despite adequate year-round sunlight for vitamin D synthesis. However, data on vitamin D status in Palestine are scarce. The aim of this cross-sectional study was to evaluate vitamin D status based on serum concentrations of 25-hydroxycholecalciferol [25-(OH)D] among young healthy Palestinian students (18–27 years) and to assess associations between 25-(OH)D concentrations and several predictors. The mean 25-(OH)D concentration of women (*n* 151) was 27⋅2 ± 14⋅5 nmol/l, with the majority having insufficient (31⋅1 %) or deficient (<60 %) 25-(OH)D status. Only 7 % of women achieved sufficient or optimal 25-(OH)D status. In contrast, men (*n* 52) had a mean 25-(OH)D concentration of 58⋅3 ± 14⋅5 nmol/l, with none classified as deficient, and most obtaining sufficient (55⋅8 %) or even optimal 25-(OH)D status (11⋅5 %). Among women, 98 % wore a hijab and 74 % regularly used sunscreen. Daily dietary vitamin D intake (3-d 24-h recalls) was 45⋅1 ± 36⋅1 IU in the total group (no sex differences). After adjustment, multiple linear regression models showed significant associations between 25-(OH)D concentrations and the use of supplements (*B* = 0⋅069; *P* = 0⋅020) and dietary vitamin D (*B* = 0⋅001; *P* = 0⋅028). In gender-stratified analysis, the association between supplement use and 25-(OH)D concentrations was significant in women (*B* = 0⋅076; *P* = 0⋅040). The vitamin D status of women in the present cohort is critical and appears to be mainly due to wearing a hijab, regular use of sunscreen and low dietary vitamin D intake. The vitamin D status of the women should be improved by taking vitamin D containing supplements or fortified foods.

## Introduction

The Palestinian Autonomous Territories including the West Bank are classified as lower-middle-income countries^(1)^. High levels of poverty due to limited income and a large family size lead to poor social conditions^(2,3)^. A complex political and economic situation^(2)^ impede coordination between health service providers and targeted fortification interventions^(4,5)^. Knowledge about nutrition and the importance of micronutrients and their role in nutrition-related diseases is limited^(6)^. Data on the supply and biostatus of specific micronutrients such as Vitamin D are limited due to a lack of nutrition studies^(1,7)^. Besides, culture and the associated choice of clothing also play a role in vitamin D status, especially among women^(2)^.

Depending on its severity and duration, vitamin D deficiency can cause diseases such as osteomalacia and rickets^(8)^. However, an insufficient vitamin D supply may also negatively affect immunity, inflammatory processes, cardiovascular diseases or obesity^(9–12)^. The prevalence of vitamin D deficiency is high worldwide^(8,9)^, including in countries in the Middle East like the Palestinian Autonomous Territories, whose geographical location (latitude) results in year-round solar radiation for endogenous vitamin D synthesis^(2,5)^. However, data on vitamin D status in adolescents and the general population are scarce. In the few studies available in the literature, 25-(OH)D concentrations were only measured in cohorts with specific diseases such as diabetes^(13)^ or among haemodialysis patients^(14)^. Studying young adults in particular in this case is of interest, as they are still before reaching their peak bone mass (PBM). Several factors, including vitamin D status, are associated with a higher bone mineral density and therefore higher PBM. This, in turn, leads to a lower risk of fractures in old age^(15–18)^.

As only a few foods (e.g. oily fish) contain vitamin D, the intake via the diet is limited. Therefore, dietary sources only cover up to 20 % of the vitamin D requirement, often less^(10,19)^. The main part of vitamin D has to be met by endogenous synthesis requiring sunlight exposure^(10)^. When the skin is exposed to sunlight, cholecalciferol (vitamin D3) is synthesised from the endogenous prohormone 7-dehydrocholesterol. In this process, the B ring of 7-dehydrocholesterol is cleaved by UV-B light radiation from the sun to form pre-D3, which is isomerised to D3 in a thermosensitive but non-catalytic process^(20,21)^. The resulting cholecalciferol is bound to vitamin D-binding proteins and metabolised in a two-step hydroxylation conversion process to 25-hydroxyvitamin D (25-(OH)D, Calcidiol) in the liver and afterwards to the active metabolite 1,25 hydroxyvitamin D (1,25-(OH)D, Calcitriol) in the kidney. In general, vitamin D synthesis is influenced by environmental factors such as season, latitude or weather conditions and individual factors such as sun exposure, sunscreen use, clothing, supplementation, skin pigmentation and age^(2,22)^. In addition, further determinants such as BMI or body fat mass and various diseases such as obesity and hyperthyroidism are known to be associated with vitamin D status. Consistent with other studies, a cross-sectional study showed that vitamin D supplementation was associated with higher serum 25-(OH)D concentrations in Saudi Arabian women^(23)^.

For these reasons, dietary vitamin D intake (excluding supplements) does not give an indication of the vitamin D status in the body. In order to determine the vitamin D status, the serum 25-(OH)D concentration is a well-established and most commonly used biomarker. This is partly due to the half-life of 3 weeks^(19,24)^. There is a debate as to what concentrations should be considered optimal, moderate, insufficient or deficient. The Institute of Medicine (IOM)^(25)^ and the European Food Safety Authority (EFSA)^(26)^ consider a concentration of 25-(OH)D > 50 nmol/l to be sufficient to meet the needs of at least 97⋅5 % of the population. However, the Endocrine Society suggests that a target concentration of at least 75 nmol/l of 25-(OH)D is sufficient^(27)^.

The aim of this cross-sectional study was to evaluate the vitamin D status in young healthy Palestinian subjects using the serum 25-(OH)D concentrations as biomarker and to characterise demographic and behavioural traits associated with these concentrations.

## Experimental methods

### Study design and ethical approvals

The present study is part of a comprehensive cross-sectional study on the health and micronutrient status among young people in the Palestinian Autonomous Territories. This study was conducted according to the guidelines laid down in the Declaration of Helsinki and all procedures involving human subjects/patients were approved by the Deanship of Scientific Research Ethical Committee at Palestine Polytechnic University (reference number KA/41/2019). Written and verbal informed consent was obtained from all subjects prior to data collection. The study population was randomly recruited at the Palestine Polytechnic University (Hebron city) by using the matriculation numbers through the help from the registration department. Students between the ages of 18 and 30 years were invited. Subjects meeting the following criteria were excluded from this study: Pregnant or breastfeeding women; participants having a celiac or inflammatory bowel disease and those rejecting to participate or refusing to sign the written consent.

### Data collection

Data collection was conducted over the course of 4 months (starting in June 2021 until the beginning of October 2021) in a face-to-face interview through four researchers using a pre-tested questionnaire including socio-demographic and nutritional data. Several questions on nutritional supplements were asked including regular use (yes [regular or irregular] *v*. no), type of supplement, duration of intake and indication. The diet intake was recorded using three 24-h recalls (two weekdays, one weekend day).

### Measurement of anthropometry

Anthropometric measurements, such as weight and height, were taken at the nutrition assessment lab at Palestine Polytechnic University – Hebron city following the standard methods reported by Lee and Niemann^(28)^. Body mass index (BMI) was calculated as bodyweight in kilograms divided by height squared in metre (kg/m^2^) and afterwards subdivided into groups according to the WHO cut-offs^(29)^. Waist circumference (WC) was measured between the lowest rib and the highest hipbone at the narrowest point of the midsection using a measuring tape. Body composition was analysed using a bioelectrical impedance analyzer (BIA) (Nutriguard M, Data Input Company, Darmstadt, Germany) and the software NutriPlus© 5.4.1 (Data Input Company, Darmstadt, Germany). The following BIA markers were evaluated: body fat (BF), lean body mass (LBM), total body water (TBW) and phase angle (PA). For the measurements, the participants were instructed to lay down on a stretcher and rest for a few minutes to ensure a balanced distribution of body fluids before the measurement. During the measurement, participants were instructed to lay still and in a relaxed position with the limbs slightly bent from the torso. A trained nutritionist carried out all measurements. All measurements were carried out in duplicate and then averaged.

### Blood collection and 25-(OH)D measurement

Blood samples were collected in the nutrition assessment lab at Palestine Polytechnic University in the morning after an overnight fast by venipuncture of an arm vein. Biochemical analyses were carried out in an external laboratory (Medicare Laboratory, Hebron city, Palestine, External Quality Control RIQAS – UK, Internal Quality Control Assayed B, 1, 2 and 3 Randox UK). Serum vitamin D concentrations were determined quantitatively in a batch analysis by chemiluminescent microparticle immunoassay (CMIA) using the Abbott Architect 25-OH vitamin D testing kit (Abbott Diagnostics, Lake Forest, IL, USA). This kit has been standardised to the standard reference material (SRM, 25-hydroxyvitamin D calibration solution) of the National Institute of Standards and Technology (NIST) (NIST SRM 2972). Samples were measured in triplicates. The coefficients of intra-assay and inter-assay variation were 1⋅93 ± 0⋅60 % (target ≤ 2⋅2 %) and 1⋅71 ± 0⋅43 % (target ≤ 3⋅3 %), respectively.

### 25-(OH)D reference ranges

The basis for assessing serum 25-(OH)D concentrations is the cut-off of >50 nmol/l for a sufficient vitamin D status^(25,26)^. Further cut-offs are established according to the classification of numerous recent publications^(5,8,11,30–33)^. 25-(OH)D concentrations > 75 nmol/l are considered as optimal, while 25-(OH)D concentrations between 25 and <50 nmol/l are considered as insufficient, and concentrations <25 nmol/l are considered as deficient.

To assess the amount of dietary vitamin D, the dietary reference values (DRVs) of 600 IU (15 μg^(25)^) and 800 IU (20 μg^(34)^) are applied. These DRVs are valid for the age group of 18–69 years to reach a 25-(OH)D target value of 50 nmol/l in conditions of minimal or no endogenous vitamin D synthesis (insufficient sunlight [UV-B] exposure)^(31)^.

### Statistical analysis

All statistical analyses were performed using SPSS software (IBM SPSS Statistics 28.0; Chicago, IL, USA). Results are presented either as mean ± standard deviation (sd) or as percentages with respect to the sample including the absolute number of cases. Independent samples *t*-tests and chi-square tests were used to evaluate differences in demographic and behavioural characteristics by sex. Linear regression models were used to assess the associations between 25-(OH)D concentrations and the same demographic and behavioural factors (sex, short sleeves (y/n/m), regular sunscreen use (y/n), BMI, WC, supplement use (y[regular and irregular]/n) and vitamin D intake (mean of the three 24-h recalls). To improve normality, the 25-(OH)D concentration data were log-transformed. Initial regression models considered each predictor's unadjusted association with vitamin D (Model 1). Subsequent models considered additional levels of covariate adjustment: Model 2 (age and sex) and Model 3 (age, sex, supplements and vitamin D use). Potential interactions between these characteristics and sex were also separately added to each model. Finally, linear regression models were fit separately for women and men, predicting 25-(OH)D by supplements and, separately, vitamin D intake. A significance threshold of 0⋅05 was used for all analyses.

## Results

### Characteristics of the study population

This cross-sectional study had a sample size of *n* 203 participants. Of these, 151 were women (74⋅4 %) and 52 (25⋅6 %) were men. 86⋅6 % of students lived at home with their parents. The demographic and anthropometric characteristics of the population are shown in Table 1; dietary and lifestyle data and clothing choices are shown in Table 2.

**Table 1.** Demographic and anthropometric characteristics of the study population

Parameter	Description	Total (*n* 203) mean ± sd or %	Female (*n* 151) mean ± sd or %	Male (*n* 52) mean ± sd or %	*P*-value (f/m)
Age (Years)		20⋅2 ± 1⋅54	20⋅3 ± 1⋅55	19⋅8 ± 1⋅43	**0⋅026**
Family income (NIS)	<1500	3⋅9 %	4⋅6 %	1⋅9 %	
	1500 to <3000	21⋅7 %	23⋅2 %	17⋅3 %	
	3000–5000	43⋅8 %	46⋅4 %	36⋅5 %	
	>5000	30⋅5 %	25⋅8 %	44⋅2 %	
Body weight (kg)		59⋅7 ± 12⋅9	55⋅6 ± 9⋅34	71⋅6 ± 14⋅3	**<0⋅001**
Height (cm)		162 ± 7⋅99	160 ± 5⋅79	172 ± 5⋅87	**<0⋅001**
BMI (kg/m^2^)		22⋅4 ± 3⋅81	21⋅8 ± 3⋅45	24⋅1 ± 4⋅31	**<0⋅001**
Underweight	<18⋅5	9⋅36 %	11⋅9 %	1⋅92 %	
Normal	18⋅5 to <25	71⋅9 %	75⋅5 %	61⋅5 %	
Overweight	25 to <30	12⋅8 %	9⋅27 %	23⋅1 %	
Obese	≥30	5⋅91 %	3⋅31 %	13⋅7 %	
WC (cm)		80⋅1 ± 9⋅09	78⋅4 ± 7⋅76	85⋅1 ± 10⋅8	**<0⋅001**
Low risk	Female: <88 Male: <102	90⋅1 %^a^	88⋅7 %^a^	94⋅2 %^a^	
High risk	Female: ≥88 Male: ≥102	9⋅85 %^a^	11⋅3 %^a^	5⋅77 %^a^	
BF (%)		19⋅6 ± 8⋅63	21⋅6 ± 8⋅28	13⋅8 ± 6⋅83	**<0⋅001**
LBM (kg)		47⋅6 ± 9⋅54	43⋅0 ± 3⋅95	61⋅0 ± 8⋅36	**<0⋅001**
TBW (kg)		34⋅8 ± 7⋅01	31⋅4 ± 2⋅89	44⋅7 ± 6⋅12	**<0⋅001**
PA		6⋅49 ± 1⋅13	6⋅28 ± 1⋅18	7⋅10 ± 0⋅68	**<0⋅001**

NIS, New Israel Shekel; BMI, body mass index; WC, Waist circumference; BF, body fat; LBM, lean body mass; TBW, total body water; PA, phase angle.

a Sex-specific cut-off values for WC (female 88 cm, male 102 cm) were used to calculate the total percentages.

**Table 2.** Further characteristics of the study population including nutritional, lifestyle and clothing data

Parameter	Description	Total (*n* 203) % (*n*)	Female (*n* 151) % (*n*)	Male (*n* 52) % (*n*)	*P*-value (f/m)
Nutritional data^a^					
Eggs (1–2 Servings/wk)	Yes	**56⋅2** % (*n* = 114)	**55⋅6 %** (*n* = 84)	**57⋅7 %** (*n* = 30)	0⋅945
	No	**41⋅9** % (*n* = 85)	**41⋅7 %** (*n* = 63)	**42⋅3 %** (*n* = 22)	
	Missing/no answer	**1⋅97** % (*n* = 4)	**2⋅65 %** (*n* = 4)	**0 %** (*n* = 0)	
Fish and seafood (≥2 servings/wk)	Yes	**9⋅4 %** (*n* = 19)	**9⋅93 %** (*n* = 15)	**7⋅69 %** (*n* = 4)	0⋅649
	No	**89⋅7 %** (*n* = 182)	**89⋅4 %** (*n* = 135)	**90⋅4 %** (*n* = 47)	
	Missing/no answer	**0⋅99 %** (*n* = 2)	**0⋅66 %** (*n* = 1)	**1⋅92 %** (*n* = 1)	
Supplements	Yes	**21⋅2 %** (*n* = 43)	**21⋅9 %** (*n* = 33)	**19⋅2 %** (*n* = 10)	0⋅781
	Regularly	***3***⋅***45 %*** (*n = 7*)	***2***⋅***65 %*** *(**n** = 4)*	***5***⋅***77 %*** *(n = 3)*	
	Irregularly	***17***⋅***7 %*** *(n = 36)*	***19***⋅***2 %*** *(n = 29)*	***13***⋅***5 %*** *(n = 7)*	
	No	**71⋅9 %** (*n* = 146)	**72⋅2 %** (*n* = 109)	**71⋅2 %** (*n* = 37)	
	Missing/no answer	**6⋅9 %** (*n* = 14)	**5⋅96 %** (*n* = 9)	**9⋅62 %** (*n* = 5)	
Lifestyle data^a^					
Smoking (cigarettes or shisha)	Yes	**14⋅3 %** (*n* = 29)	**11⋅3 %** (n = 17)	**23⋅1 %** (*n* = 12)	**0⋅045**
	Regularly	***2***⋅***46 %*** *(n = 5)*	***0***⋅***66 %*** *(n = 1)*	***7*⋅*****69 %** (n = 4)*	
	Irregularly	***11***⋅***8 %*** *(n = 24)*	***10***⋅***6 %*** *(n = 16)*	***15***⋅***4 %*** *(n = 8)*	
	No	**83⋅3 %** (*n* = 169)	**85⋅4 %** (*n* = 129)	**76⋅9 %** (*n* = 40)	
	Missing/no answer	**2⋅46 %** (*n* = 5)	**3⋅31 %** (*n* = 5)	**0 %** (*n* = 0)	
Sun exposure (go for a walk)	Yes	**45⋅8 %** (*n* = 93)	**46⋅4 %** (*n* = 70)	**44⋅2 %** (*n* = 23)	0⋅816
	No	**52⋅7 %** (*n* = 107)	**52⋅3 %** (*n* = 79)	**53⋅8 %** (*n* = 28)	
	Missing/no answer	**1⋅48 %** (*n* = 3)	**1⋅32 %** (*n* = 2)	**1⋅92 %** (*n* = 1)	
Sunscreen (regular usage)	Yes	**55⋅2 %** (*n* = 112)	**73⋅5 %** (*n* = 111)	**1⋅82 %** (*n* = 1)	**<0⋅001**
	No	**39⋅9 %** (*n* = 81)	**24⋅5 %** (*n* = 37)	**84⋅6 %** (*n* = 44)	
	Missing/no answer	**4⋅93** % (*n* = 10)	**1⋅99 %** (*n* = 3)	**13⋅5 %** (*n* = 7)	
Living area	City	**71⋅9** % (*n* = 146)	**76⋅2 %** (*n* = 115)	**59⋅6 %** (*n* = 31)	0⋅071
	Village	**25⋅1** % (*n* = 51)	**21⋅2 %** (*n* = 32)	**36⋅5 %** (*n* = 19)	
	Camp	**2⋅96** % (*n* = 6)	**2⋅65 %** (*n* = 4)	**3⋅85 %** (*n* = 2)	
Clothing data^a^					
Hijab^b^	Yes		**98⋅0 %** (*n* = 148)		–
	No		**1⋅32 %** (*n* = 2)		
	Missing/no answer		**0⋅66 %** (*n* = 1)		
Niqab^c^	Yes		**0⋅66 %** (*n* = 1)		–
	No		**98⋅0 %** (*n* = 148)		
	Missing/no answer		**1⋅32 %** (*n* = 2)		
Short sleeves	Yes	**12⋅8 %** (*n* = 26)	**5⋅96 %** (*n* = 9)	**32⋅7 %** (*n* = 17)	**<0⋅001**
	No	**75⋅4 %** (*n* = 153)	**92⋅7 %** (*n* = 140)	**25⋅0 %** (*n* = 13)	
	Missing/no answer	**11⋅8 %** (*n* = 24)	**1⋅32 %** (*n* = 2)	**42⋅3 %** (*n* = 22)	
Short skirt	Yes		**0 %** (*n* = 0)		–
	No		**97⋅4 %** (*n* = 147)		
	Missing/no answer		**2⋅65 %** (*n* = 4)		

a Multiple selection possible.

b Hijab: Cloth that covers the hair and neck but leaves the face visible.

c Niqab: Cloth that covers the hair, neck and the face, but leaves the eye area uncovered.

The queried and measured metrics assessing basic health indicate a relatively young (mean age of 20⋅2 ± 1⋅54 years; range 18–27 years) and reasonably healthy sample, as predictors considered to be risk factors for various diseases (e.g. cardiovascular disease, metabolic syndrome), such as a high BMI and WC, or poorer living conditions due to a lower family income or regular smoking, are hardly present (Table 1). All characteristics showed statistically significant differences between women and men (*P* < 0⋅05). Most women were within the normal BMI range. Only 9⋅27 and 3⋅31 % are overweight and obese, respectively. When looking at the WC, however, it is noticeable that 11⋅3 % of all women were in the high-risk range. The situation is different for men. More than a third were overweight or obese. However, the proportion of men with a WC at the highest risk level for cardiovascular disease is only 5⋅77 %.

Slightly more than half of the study population consumed 1–2 eggs/wk, whereas less than 10 % reported a regular fish consumption of ≥2 servings/wk (Table 2). Only about a fifth of the participants reported taking supplements, but most of them reported doing so irregularly. Unfortunately, subjects that indicated regular supplement use did not provide further or precise information on the supplements used.

Some differences between men and women concerning sunscreen use and clothing were striking: approximately three quarters of women stated that they regularly used sunscreen, while the proportion of men reporting use was under 2 %. The vast majority of women wore a hijab (98 %) and, thus, due to the confounding of sex with hijab use, hijab was not included in any further analyses. Since only one woman stated wearing a Niqab, this variable was not analysed in the further course of this study. Only 5⋅96 % of women stated wearing clothes with short sleeves, while none of the women wore short skirts. In contrast, 32⋅7 % of the men said they wore short sleeves, however, the percent of men who skipped this question was very high (42⋅3 %).

93⋅1 % of the female participants had a vitamin D intake below 100 IU and just 6⋅9 % had a vitamin D intake of 100–200 IU (Table 3). This meant that no one in the sample reached the recommended vitamin D intake of at least 600 IU. Although men had higher vitamin D intake, the difference between men and women was not statistically significant.

**Table 3.** Daily vitamin D intake calculated from three separate 24-h recalls

Parameter	Total (n 195) mean ± sd % (*n*)	Female (*n* 151) mean ± sd % (*n*)	Male (*n* 51) mean ± sd % (*n*)	*P*-value (f/m)
Vitamin D intake (IU)	**45⋅1 ± 36⋅1**	**42⋅8 ± 35⋅3**	**51⋅6 ± 37⋅9**	0⋅138
>200	**0 %**	**0 %**	**0 %**	0⋅831
100–200	**7⋅2 %** (n = 14)	**6⋅9 %** (*n* = 10)	**7⋅8 %** (*n* = 4)	
<100	**92⋅8 %** (n = 181)	**93⋅1 %** (*n* = 134)	**92⋅2 %** (*n* = 47)	

### Serum 25-(OH)D concentrations

**Fig. 1.**
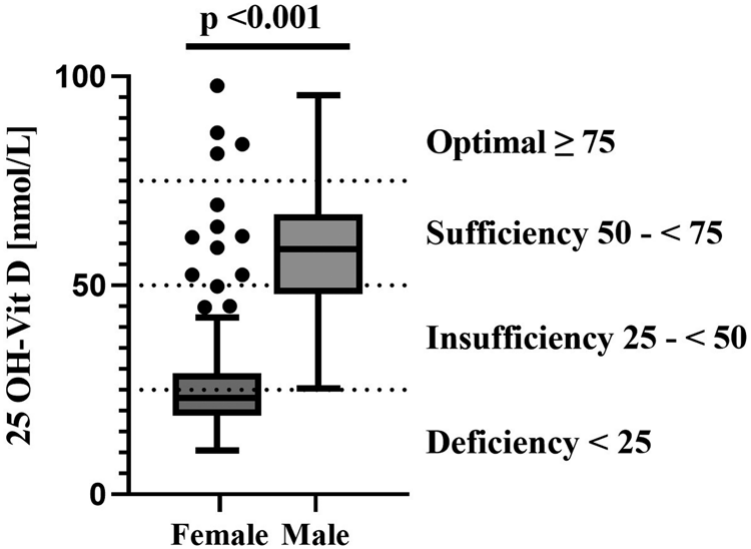
Serum 25-(OH)D concentrations in females and males.

The mean 25-(OH)D concentration ± the standard deviation (sd) in the total study population was 35⋅2 ± 19⋅8 nmol/l, however, men had concentrations approximately twice as high (58⋅3 ± 14⋅5 nmol/l) as women (27⋅2 ± 14⋅5 nmol/l, Fig. 1), leading to a significant difference (*P* < 0⋅001) between women and men. The majority of women had insufficient (31⋅1 %) or even deficient (<60 %) serum 25-(OH)D concentrations (Table 4). Only about 7 % of women showed 25-(OH)D concentrations that indicate a sufficient or optimal vitamin D status. In contrast, none of the men were in a deficient vitamin D status range, most men had sufficient (55⋅8 %) or optimal (11⋅5 %) vitamin D concentrations.

**Table 4.** Serum 25-(OH)D concentrations by subcategories

Description	Total (*n* = 203) mean ± sd (%, *n*)	Female (*n* = 151) mean ± sd (%, *n*)	Male (*n* = 52) mean ± sd (%, *n*)
Optimal ≥75 (nmol/l)	**86⋅1 ± 6⋅59** (4⋅93 %, *n* = 10)	**87⋅4 ± 7⋅21** (2⋅65 %, *n* = 4)	**85⋅3 ± 6⋅69** (11⋅5 %, *n* = 6)
Sufficient 50–75 (nmol/l)	**61⋅3 ± 6⋅21** (17⋅7 %, *n* = 36)	**60⋅1 ± 6⋅06** (4⋅64 %, *n* = 7)	**61⋅6 ± 6⋅31** (55⋅8 %, *n* = 29)
Insufficient 25 to <50 (nmol/l)	**35⋅0 ± 7⋅98** (31⋅5 %, *n* = 64)	**32⋅1 ± 6⋅22** (31⋅1 %, *n* = 47)	**43⋅1 ± 6⋅78** (32⋅7 %, *n* = 17)
Deficient <25 (nmol/l)	**19⋅7 ± 3⋅17** (45⋅8 %, *n* = 93)	**19⋅7 ± 3⋅17** (61⋅6 %, *n* = 93)	(0 %, *n* = 0)

With the exception of ‘supplements’, all other measures included in the linear regression analyses were statistically significantly associated with the serum 25-(OH)D concentrations in unadjusted models (Model 1; Table 5). The association was particularly strong for the association of sex with 25-(OH)D concentrations. Regular sunscreen users had lower 25-(OH)D concentrations. For all other independent variables, the association with the 25-(OH)D concentrations was less pronounced.

**Table 5.** Multiple linear regression models to examine associations of serum 25-(OH)D levels with various predictors in the total study population and in sex-stratified analysis

Parameter	Model 1^a^			Model 2^b^		Model 3^c^		Interaction *P*-value*
	*R*^2^	*B*	*P*-value	*B*	*P*-value	*B*	*P*-value	
Entire sample								
Sex (*n* = 203)^d^	0⋅485	0⋅358	**<0⋅001**	0⋅352^e^	**<0⋅001**	0⋅348^e^	**<0⋅001**	–
Age (*n* = 203)^d^	0⋅033	−0⋅027	**0**⋅**009**	−0⋅011^f^	0⋅141	0⋅021^f^	0⋅858	0⋅45
Short sleeves (*n* = 203)^d^	0⋅242	0⋅161	**<0⋅001**	0⋅003	0⋅903	0⋅003	0⋅915	0⋅35
Regular sunscreen use (*n* = 193)^d^	0⋅149	−0⋅171	**<0⋅001**	0⋅031	0⋅302	0⋅025	0⋅421	0⋅50
BMI (*n* = 203)^d^	0⋅027	0⋅010	**0**⋅**018**	−0⋅001	0⋅682	−0⋅001	0⋅804	0⋅31
WC (*n* = 203)^d^	0⋅027	0⋅004	**0**⋅**019**	−0⋅002	0⋅191	−0⋅002	0⋅216	0⋅66
Supplements (*n* = 189)^d^	0⋅013	0⋅061	0⋅120	0⋅066	**0**⋅**022**	0⋅069	**0**⋅**020**	0⋅48
Vitamin D intake (*n* = 195)^d^	0⋅031	0⋅001	**0**⋅**014**	0⋅001	**0**⋅**026**	0⋅001	**0**⋅**028**	0⋅74
Females only^e^								
Supplements (*n* = 142)^d^	0⋅036	0⋅080	**0**⋅**024**	0⋅013	**0**⋅**030**	0⋅076	**0**⋅**040**	–
Vitamin D intake (*n* = 144)^d^	0⋅020	0⋅001	0⋅089	0⋅001	0⋅060	0⋅001	0⋅080	–
Males only^e^								
Supplements (*n* = 47)^d^	0⋅014	0⋅033	0⋅432	0⋅032	0⋅446	0⋅045	0⋅303	–
Vitamin D intake (*n* = 51)^d^	0⋅025	<0⋅001	0⋅267	0⋅001	0⋅244	0⋅001	0⋅251	–

BMI, body mass index; WC, waist circumference.

Due to high number of missing data from questionnaires, linear regression for ‘short sleeves’ was performed with the categories yes/no/missing, while analysis for ‘regular sunscreen use’ and ‘supplements’ were performed with binary categories yes/no.

a Before adjustment.

b Adjusted for sex and age.

c Adjusted for sex, age, supplements and vitamin D intake.

^d^*n* defines the number of complete data included in the respective analysis.

^e^Not adjusted by sex.

f Not adjusted by age.

* Testing for interaction between each characteristic and sex in Model 1 when predicting 25-(OH)D concentrations.

After adjusting each model by sex and age (Model 2) and, additionally by supplements and vitamin D intake (Model 3), numerous variables showing unadjusted associations with vitamin D concentration were no longer significantly associated with the 25-(OH)D concentration. The exceptions were sex, vitamin D intake and supplement taking, which each continued to be significantly associated with 25-(OH)D concentrations even after adjusting for other variables.

Sex-stratified analyses were conducted for supplements and vitamin D intake, revealing a statistically significant association between supplements and 25-(OH)D concentrations in women, but not in men. This association remained significant in women even after adjustments in Models 2 and 3.

However, testing for interaction between each characteristic and sex in Model 1 of the linear regression when predicting 25-(OH)D concentrations of interaction with sex were not statistically significant (*P* > 0⋅05).

## Discussion

Palestine is geographically located in a region with sufficient sunlight throughout the year, potentially providing enough sunlight to allow for sufficient endogenous synthesis of Vitamin D^(2,5)^. Nevertheless, this cross-sectional study shows that the 25-(OH)D concentrations among female Palestinian students is alarmingly poor: 31⋅1 % of the women had an insufficient vitamin D status, while 61⋅6 % had a vitamin D deficiency. On the other hand, mean 25-(OH)D concentrations were twice as high in men as in women. None of the male participants had a deficient vitamin D status. A similar poor vitamin D status was observed in surrounding countries or those with similar socio-cultural background and sufficient sunlight irradiation^(1,35–39)^. In Iran and Pakistan, 51 and 58 % of adults, respectively, had had 25-(OH)D concentrations <50 nmol/l, with no further differentiation for women and men^(36,39)^. Studies from Jordan and Israel showed differentiated results for both sexes. In Jordan, 14 % of the women and only 2 % of men had vitamin D blood concentrations <50 nmol/l^(35)^. Of the male subjects in Israel, 9–12 % had deficient, 32–38 % insufficient and 34–36 % sufficient vitamin D status^(38)^. Among women, 15–23 % were deficient, 36–37 % were insufficient and 28–29 % sufficient, depending on the season (winter/spring or summer/autumn). Pregnant and breastfeeding women in Kuwait showed 25-(OH)D concentrations, which were in an insufficient range in 32–42 % and in a deficient range in 38–41 %^(37)^.

Vitamin D supply is a problem worldwide. Even in the USA or European countries, the prevalence of vitamin D deficiency is high^(6)^. However, the situation is not as dramatic as in Palestine. The vitamin D status of the female participants in the existing cohort is significantly lower than the vitamin D status of women in Western European countries^(40)^. For example, a German study with 429 non-pregnant women showed that 54 % of the women were in an insufficient vitamin D range, while only 13 % were in a deficient range^(10)^. In two other studies that were carried out in Estonia (*n* 367) and Norway (*n* 4465), 1 % of the Estonian subjects had deficient and 29 % insufficient 25-(OH)D concentrations in summer with equal distribution of sex^(41)^, while 2 % of the Norwegian subjects had deficient and 25 % insufficient 25-(OH)D concentrations for both sexes^(42)^.

Although the vitamin D requirement is mainly (approx. 80–90 %) covered through endogenous synthesis in the skin, the remaining 10–20 % comes from the diet^(10)^. Dependent on the expert societies, the recommended daily vitamin D intake is between 600 IU^(25)^ and 800 IU^(34)^. Less than 10 % of both women and men in our study cohort reached a vitamin D intake of 100–200 IU daily, while none of the participants had a vitamin D intake of >200 IU. More than 90 % had a daily vitamin D intake of <100 IU. Our findings are also reflected in other studies in the Middle East^(30)^. In a study in Kuwait (*n* 1049), the vitamin D intake ranged from 40 to 80 IU^(43)^, while in a study in Qatar (60 women), the mean vitamin D intake was 120 IU^(44)^. In comparison, studies in many Western European countries observe a significantly higher vitamin D intake. Jenab *et al.* showed that the mean vitamin D intake for the southern part of Europe were 168 IU for men and 204 IU for women, while the intake in central Europe were 188 for men and 136 IU for women^(45)^. Despite the lower solar radiation, especially Nordic European countries showed a high vitamin D intake (>400 IU) due to their high (oily) fish consumption, resulting in sufficient 25-(OH)D concentrations >50 nmol/l^(40)^. In the existing cohort, 90 % of the men and women consumed less than 1 serving of fish per week, which is well below the general recommendation of at least 2 servings per week.

Many factors influence the endogenous synthesis of vitamin D and are therefore considered predictors of vitamin D status^(2,22)^. An influence of factors such as season, latitude and weather conditions as well as skin pigmentation on differences within the study population can be excluded, since these factors were similar for all participants. However, several significant associations were found between the vitamin D concentrations and factors such as sex, age, BMI, WC, vitamin D intake and sunlight exposure. Sex showed the strongest associations with vitamin D concentrations, even after adjustment for numerous confounders. A different diet can be ruled out as an influencing factor since no significant differences in vitamin D intake from food or in the frequency of fish consumption were found between men and women. The reason for the sex-specific differences appears to be largely due to clothing and hence sunlight exposure.

The clothing style of the women in Palestine is predominantly religious. Almost all women in the present cohort reported wearing a *hijab* in combination with dresses that covered legs and arms. Hence, it can be presumed that this clothing type prevents sufficient exposure to sunlight and endogenous vitamin D synthesis. The influence of wearing a hijab cannot be statistically proved in this cohort, due to the confounding sex and hijab status. Almost all women wore a hijab in combination with leg and arm covering clothing and no ‘control’ group of women was available who did not wear this clothing type. Moreover, the 25-(OH)D concentrations of women were in a very narrow low range. Similar difficulties in the investigation of this relationship were obvious in a study with healthy premenopausal women in Kuwait^(46)^. Even if the authors did not observed a significant association of wearing a hijab and 25-(OH)D concentrations, since the overall vitamin D concentrations were extremely low, a trend was observed that women with western clothing's choice had higher vitamin D concentrations than those with a hijab (face and hands uncovered) or those with a completely veiled body^(46)^. However, a study in Bahrein, observed that vitamin D deficiency in women increased significantly with conservative clothing styles^(47)^.

Furthermore, we see associations between the *use of sunscreen* and the vitamin D concentrations, which, however, was no longer significant after correction for various confounders. Findings in the literature on the role of sunscreen use for vitamin D blood concentrations are inconsistent. In a systematic meta-analysis, only few associations were found between sunscreen use and lower vitamin D concentrations^(48)^. However, underlying studies are difficult to compare because of differences in the sun protection factor and thickness of sunscreen application. In addition, no placebo-controlled studies are available to support the hypothesised effect. There is also a discrepancy between the advantage of vitamin D synthesis without sunscreen and the disadvantage of being unprotected against possible skin cancer. Overall, sunscreen use also suggests that people spend time in the sun, which in turn results in higher concentrations^(49)^.

In the cross-sectional study of AlFaris *et al.*, with 168 female participants in Riyadh (Saudi Arabia), the use of *supplements* was observed as a major predictor. Women taking vitamin D, multivitamin or calcium supplements tend to have higher vitamin D status^(23)^. Our data confirm these findings, but only for women. Even after adjusting the female-specific regression analysis with age and vitamin D intake, there is still a significant association between supplement use and vitamin D concentrations. Due to the lack of information from the supplement-users about the preparations used, no statements can be made if vitamin D containing supplements were used. We can only speculate whether the reason for the lack of association between supplement use and vitamin D concentrations in men is due to higher sun exposure (not wearing a hijab, less sunscreen use). In any case, the intake of vitamin D via the diet (as well as the consumption of fish and supplements) was comparable in men and women. Cross-sectional studies in Saudi Arabia^(50)^ and Bahrain^(47)^ found a higher prevalence of vitamin D deficiency in younger women, which the authors attributed to an unhealthy diet (e.g. high fast food consumption) and low use of vitamin supplements. This may apply to some extent to our cohort. However, the situation in Palestine is exacerbated by the fact that fish and supplements are poorly available and expensive and therefore rarely used.

An adequate vitamin D status is important for the prevention of different diseases associated with a 25-(OH)D deficiency e.g. osteomalacia and rickets^(8)^. In addition, associations with insufficient vitamin D status and immunity, inflammatory processes, cardiovascular diseases or obesity are currently investigated^(9–12)^.

Especially for women's health, vitamin D plays an important role. The vitamin is associated with breast cancer risk, fertility, diseases such as endometriosis or polycystic ovary syndrome (PCOS) and hormone synthesis related to the menstrual cycle^(51)^. With an insufficient or even deficient vitamin D status – which is the case for most of the female participants in our study – the risk for female health-specific diseases is increased^(51,52)^. Also, in relation to pregnancy and breastfeeding, a vitamin D deficiency can impact the fetal and maternal health of the fetus or the mother, such as reduced bone mineral content, acute respiratory tract infections, preeclampsia, gestational diabetes, premature birth and fetal growth disorders^(19)^.

Also, for men's health, a sufficient vitamin D status is important. An insufficient or deficient vitamin D status is associated with infertility and poorer semen quality^(53)^. The meta-analysis of Arab *et al.* shows a significant association between vitamin D status and fertility. In addition, significant associations were found with respect to sperm motility and progressive motility. No effect on semen quality was found, but this can also be influenced by other confounding factors^(53)^. However, in the present cohort, none of the men had a vitamin D status in the deficient range.

## Limitations

The present study has several limitations. The study cohort has overall a relatively small sample size, which are all within an age range of 18–27 and therefore results cannot be extrapolated to the general population of Palestine. Because the original study was designed to investigate women's health only, the comparison group (men) has a smaller number of cases, limiting power to detect sex differences. In addition to that, a possible bias due to the cultural norm of the choice of clothing is conceivable. The calculation of vitamin D intake from diet-based 24-h recalls is inexact due to over- and under-reporting of food intake, inaccurate information on vitamin D concentrations in food databases as well as variations due to cooking methods, thus our focus on serum 25-(OH)D concentrations analysed using CMIA is a strength. However, we note that CMIA measurement may overestimate 25-(OH)D at high concentrations compared with high-pressure liquid chromatography combined with mass spectrometric analyses (LC-MS/MS)^(54,55)^. Nevertheless, because the 25-(OH)D concentrations measured in this study were generally low, especially for women an overestimation of the 25-(OH)D concentrations is unlikely. Rather, both methods provide sufficient sensitivity to detect a vitamin D deficiency^(24,55)^.

## Conclusion

Our study showed that more than half of the young healthy female students in our study in Palestine were severely vitamin D deficient, while the men had a sufficient vitamin D status. The poor vitamin D status in women is most likely the result of an inadequate sunlight exposure due to skin veiling by wearing a hijab in combination with leg and arm covering clothing. However, further research is needed to investigate the effect of clothing (particularly the hijab) on blood vitamin D concentrations. The vitamin D status of young Palestinian women should be significantly improved to maintain health and fertility. The finding that supplement use in women was independently associated with vitamin D concentration suggests that women may be particularly likely to benefit from supplementation to improve their vitamin D status.
